# Metabolism of Toxic Sugars by Strains of the Bee Gut Symbiont *Gilliamella apicola*

**DOI:** 10.1128/mBio.01326-16

**Published:** 2016-11-01

**Authors:** Hao Zheng, Alex Nishida, Waldan K. Kwong, Hauke Koch, Philipp Engel, Margaret I. Steele, Nancy A. Moran

**Affiliations:** Department of Integrative Biology, University of Texas at Austin, Austin, Texas, USA

## Abstract

Social bees collect carbohydrate-rich food to support their colonies, and yet, certain carbohydrates present in their diet or produced through the breakdown of pollen are toxic to bees. The gut microbiota of social bees is dominated by a few core bacterial species, including the Gram-negative species *Gilliamella apicola*. We isolated 42 strains of *G. apicola* from guts of honey bees and bumble bees and sequenced their genomes. All of the *G. apicola* strains share high 16S rRNA gene similarity, but they vary extensively in gene repertoires related to carbohydrate metabolism. Predicted abilities to utilize different sugars were verified experimentally. Some strains can utilize mannose, arabinose, xylose, or rhamnose (monosaccharides that can cause toxicity in bees) as their sole carbon and energy source. All of the *G. apicola* strains possess a *manO*-associated mannose family phosphotransferase system; phylogenetic analyses suggest that this was acquired from *Firmicutes* through horizontal gene transfer. The metabolism of mannose is specifically dependent on the presence of mannose-6-phosphate isomerase (MPI). Neither growth rates nor the utilization of glucose and fructose are affected in the presence of mannose when the gene encoding MPI is absent from the genome, suggesting that mannose is not taken up by *G. apicola* strains which harbor the phosphotransferase system but do not encode the MPI. Given their ability to simultaneously utilize glucose, fructose, and mannose, as well as the ability of many strains to break down other potentially toxic carbohydrates, *G. apicola* bacteria may have key roles in improving dietary tolerances and maintaining the health of their bee hosts.

## IMPORTANCE

Bees are important pollinators of agricultural plants. Our study documents the ability of *Gilliamella apicola*, a dominant gut bacterium in honey bees and bumble bees, to utilize several sugars that are harmful to bee hosts. Using genome sequencing and growth assays, we found that the ability to metabolize certain toxic carbohydrates is directly correlated with the presence of their respective degradation pathways, indicating that metabolic potential can be accurately predicted from genomic data in these gut symbionts. Strains vary considerably in their range of utilizable carbohydrates, which likely reflects historical horizontal gene transfer and gene deletion events. Unlike their bee hosts, *G. apicola* bacteria are not detrimentally affected by growth on mannose-containing medium, even in strains that cannot metabolize this sugar. These results suggest that *G. apicola* may be an important player in modulating nutrition in the bee gut, with ultimate effects on host health.

## INTRODUCTION

Gut bacteria possess a large repertoire of metabolic capabilities, and they play an important role in the fermentation of complex dietary carbohydrates in the intestines of insects, herbivorous vertebrates, and humans (1). Since animal hosts lack the enzymes to degrade most types of carbohydrates (2), these recalcitrant nutrients are often left to be digested by the intestinal microbiota, which, in turn, release fermentation products (i.e., short-chain fatty acids) that can be highly beneficial for host energy metabolism (3). For honey bees and bumble bees, foraging workers collect nectar for the carbohydrate needs of the bee colony, as well as pollen, which contains various carbohydrates, proteins, lipids, and other micronutrients that are critical for bee development and reproduction (4, 5). However, these food sources are not completely innocuous, as they may contain xenobiotics that are produced by plants or introduced during beekeeping practices (6).

The impact of carbohydrates on bee survival has been studied for nearly a century (7, 8), and it is well established that bees live longest on syrup containing sucrose, glucose, or fructose (9). Other sugars from natural nectar can exhibit strong toxicity, including the monosaccharides mannose (10), xylose, arabinose, and rhamnose, as well as some oligosaccharides; these can reduce the life span of adult bees at concentrations as low as 2% (11). Moreover, it has been shown that, during the process of pollen breakdown, hydrolysis of pectin produces a mixture of sugars that are toxic to bees (12).

Both honey bees (genus *Apis*) and bumble bees (genus *Bombus*) harbor characteristic gut microbial communities that are unique to social bees (13). The most predominant fermentative bacteria in the gut of bees are the Gram-negative species *Gilliamella apicola* (class *Gammaproteobacteria*, order *Orbales*) and the Gram-positive *Lactobacillus* species phylotypes Firm-4 and Firm-5 (14). It is believed that the gut microbiota play essential roles in honey bee and bumble bee health, with potential effects on pathogen defense and nutrient acquisition (15). *G. apicola* strains have complete glycolysis pathways in their genomes, as well as numerous genes encoding phosphotransferase systems (PTSs) (16), thus implicating their function as saccharolytic fermenters that participate in the digestion of the host’s carbohydrate-rich diet. In a metagenomic analysis, Engel et al. (17) identified genes encoding pectin-degrading enzymes in *G. apicola* that may help in the breakdown of the rigid polysaccharide walls of pollen grains and in the release of constituent monosaccharides. However, it remains unclear whether the gut bacteria can utilize these monosaccharides, some of which are toxic to the bee host.

In this study, we isolated 42 *G. apicola* strains from the guts of both honey bees and bumble bees and sequenced their genomes to assess their gene repertoires related to sugar utilization. The metabolism of specific carbohydrates was confirmed physiologically, and the ability to simultaneously utilize different sugars was determined. We analyzed genes for carbohydrate utilization in a phylogenetic framework and discovered that *G. apicola* has likely taken up relevant genes from species of *Firmicutes*. There was also substantial variation in sugar degradation capabilities among strains, suggesting niche differentiation or specialization to particular host species.

## RESULTS

### Whole-genome-based phylogeny.

We isolated 15 *G. apicola* strains from the gut homogenates of various honey bee species and 27 from bumble bee species, and the genomes of these isolates were then sequenced using the Illumina MiSeq platform (see Table S1 in the supplemental material). A complete genome of strain wkB7 was obtained using both the Illumina MiSeq and PacBio RS II platforms (see Materials and Methods). The *G. apicola* genomes range from 2.1 to 3.1 Mb in size, and the 16S rRNA gene sequences show high levels of similarity among all strains (overall mean similarity of 98.5%) (see Table S1). Nonetheless, the strains show considerable divergence at other loci, and the mean pairwise average nucleotide identity between orthologous genomic regions is only 83.0% (range, 77.9 to 99.9%).

**FIG 1:**
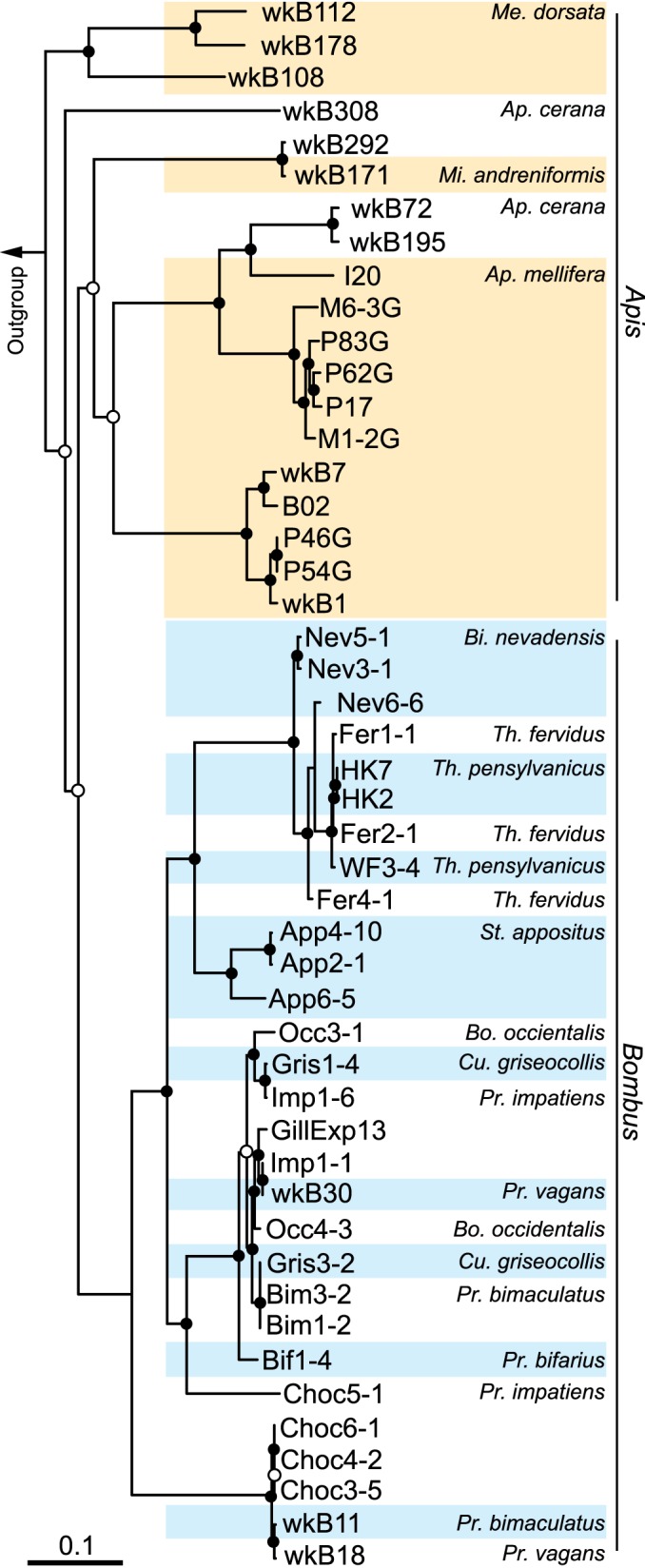
Phylogeny of 48 strains of *Gilliamella apicola* from guts of honey bees and bumble bees. Six previously published genomes (16, 18) and 42 newly sequenced genomes were included in the analysis. The tree was built using the maximum-likelihood algorithm based on the concatenated sequences of 225 single-copy genes (22,315 nucleotide positions) and rooted with sequences from *Frischella perrara* (51) and “*Candidatus* Schmidhempelia bombi” (52). Circles indicate node bootstrap support (○, >85%; ●, 100%, 1,000 replicates). Scale bar indicates 0.1 nucleotide substitutions per site. The subgenera of honey bee and bumble bee hosts are as follows: Me, *Megapis*; Mi, *Micrapis*; Ap, *Apis*; Bi, *Bombias*; Th, *Thoracobombus*; St, *Subterraneobombus*; Pr, *Pyrobombus*; Cu, *Cullumanobombus*; Bo, *Bombus*.

The genome sequences of these 42 *G. apicola* strains were then analyzed along with 6 previously published *G. apicola* genomes (16, 18). We identified a set of 225 orthologous genes present in all genomes. Phylogenetic analysis based on these shared orthologs revealed that the genomes from bumble bees clustered exclusively from those from honey bees (Fig. 1). The bumble bee-derived *G. apicola* strains appear to be monophyletic, while honey bee-derived strains were paraphyletic. Although isolates from the same honey bee species tend to cluster together, strains from *Apis dorsata* and *Apis cerana* are sisters to other *G. apicola* strains in the tree, supporting the occurrence of some host switching rather than strict host-microbe codiversification. Notably, the strains from the same subgenera of bumble bees are more closely related in the tree, and the strains from *Pyrobombus*, *Cullumanobombus*, and *Bombus* formed a separate cluster corresponding to a cluster within the phylogeny based on host DNA sequences (19).

### Evolution of mannose metabolism-related genes.

To investigate the ability of *G. apicola* to digest mannose, we identified the genes related to mannose metabolism in the genomes. In bacteria, the utilization of mannose typically requires two components: a PTS to take up extracellular mannose and mannose-6-phosphate (mannose-6-P) isomerase (MPI) to enable its catabolism via glycolysis pathways (20). The PTS is usually composed of enzyme I and HPr, which are general for all carbohydrates, and the substrate-specific enzyme II (EII) (21). EII complexes typically consist of three protein domains (IIA to -C), while the EII of the mannose PTS family is unique in having an additional IID domain (22).

**FIG 2:**
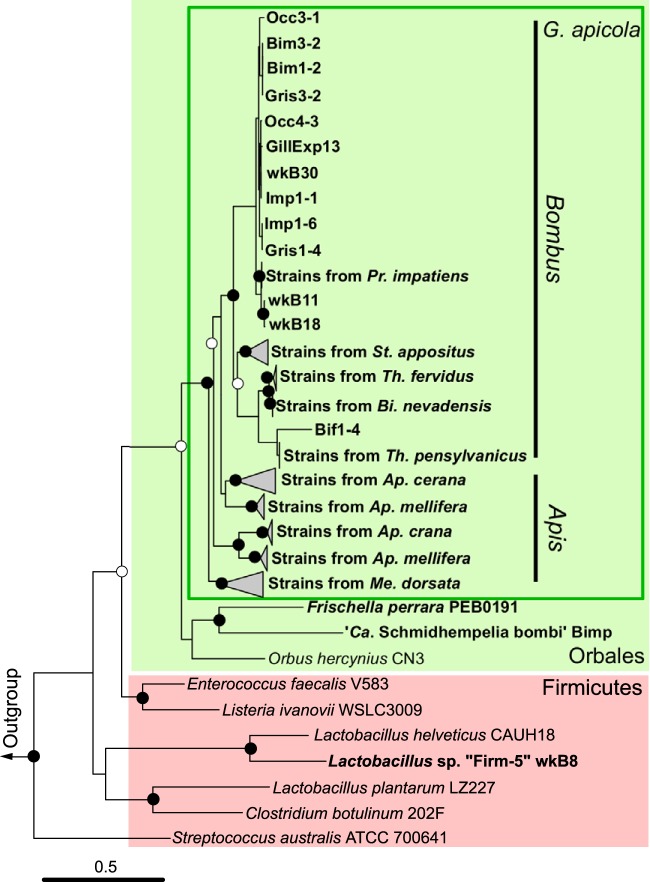
Maximum-likelihood phylogeny of the *manO*-associated mannose family PTS from *G. apicola* strains. The tree is based on the concatenated nucleotide sequences of three enzyme II genes (encoding EIIA/-B, -C, and -D). The tree was rooted with sequences from *Escherichia coli* and *Klebsiella pneumoniae*. Sequences from strains isolated from bee guts are shown in boldface. Sequences from the same host species are clustered in clades. Circles indicate node bootstrap support (○, >70%; ●, >95%). Scale bar indicates 0.5 nucleotide substitutions per site.

We identified 11 EII complexes that have IID domains in the genomes of *G. apicola* strains (see Fig. S1 in the supplemental material); one of these is composed of a fused IIA/B domain and a downstream *manO* gene and is the only EII complex present in all *G. apicola* strains. The *manO* gene is a putative regulator of the mannose EII operon and, interestingly, has only been found in *Firmicutes* until now (23). Phylogenetic analysis of the *manO*-associated mannose EII operon revealed that it is also present in relatives of *G. apicola* within the order *Orbales* (Fig. 2). All other closely related sequences were from *Firmicutes*, suggesting that this PTS system, together with *manO*, was horizontally transferred from a Gram-positive bacterium to the common ancestor of the Gram-negative *Orbales* clade.

**FIG 3:**
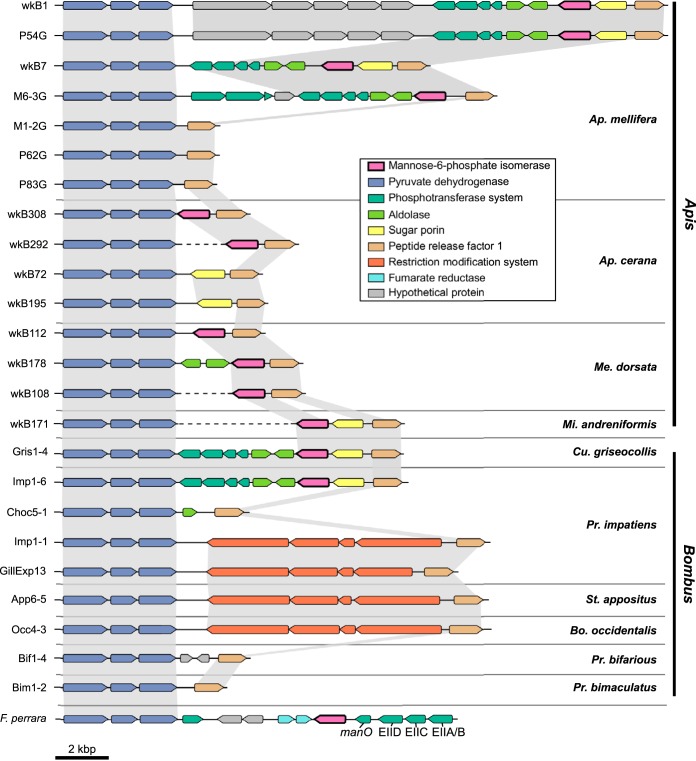
The variable region containing the mannose-6-phosphate isomerase gene (*manA*) among *G. apicola* isolates from honey bees (*Apis*) and bumble bees (*Bombus*), and *F. perrara*. The variable region extends between the genes for pyruvate dehydrogenase and peptide release factor 1. Gray shading indicates orthologous regions. Dashed lines indicate that genes are present on different contigs. The subgenera of bee hosts are as listed in the legend to Fig. 1.

In contrast, the MPI gene (*manA*) was only present in 10 strains isolated from honey bees and two strains from bumble bees (Fig. 3). In the type strain wkB1, isolated from *Apis mellifera* (16), *manA* is flanked by genes encoding pyruvate dehydrogenase (PDH), PTS EIIA to -D, aldolase, a sugar porin, and peptide release factor 1 (RF1), together with several hypothetical genes (Fig. 3). In 23 of the newly sequenced genomes, the PDH and RF1 genes were detected in the same contig and seem to bracket a variable region that, in some genomes, contains *manA*. In four strains from bumble bees (strains Imp1-6, GillExp13, App6-5, and Occ4-3), restriction-modification system genes occupy this variable region instead of the typical genes. In the genomes of strains wkB292, wkB108, and wkB171, *manA* is also found next to the RF1 gene; however, the corresponding PDH genes are located on different contigs (due to incomplete genomic assembly). Intriguingly, the *manA* gene is also present in *Frischella perrara*, another core gut bacterium isolated from *A. mellifera* (24), where it appears to have become genetically linked with the *manO*-associated EII complex (Fig. 3).

**FIG 4:**
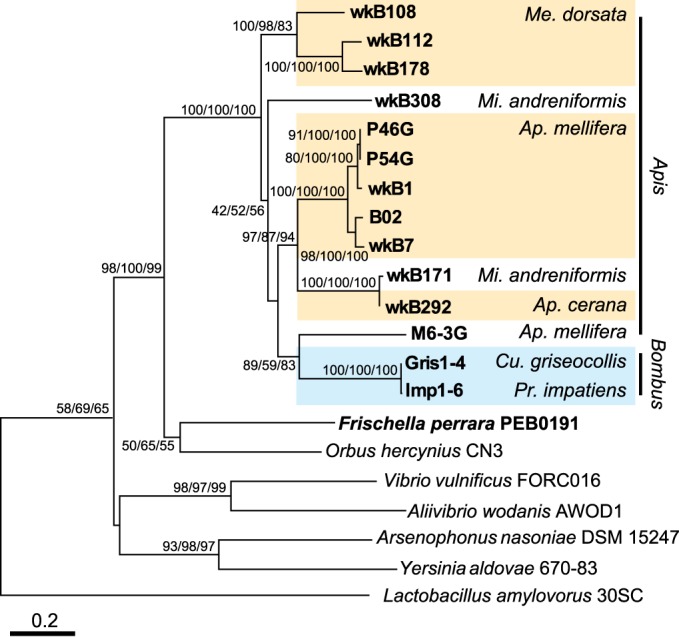
Phylogeny of the mannose-6-phosphate isomerase gene (*manA*). The tree is inferred from neighbor-joining/maximum-parsimony/maximum-likelihood methods based on nucleotide sequences and was rooted with the sequence from *Lactobacillus amylovorus*. Sequences from strains isolated from bee guts are shown in boldface. The numbers at nodes represent bootstrap confidence values (%) for each phylogeny-testing method. Scale bar indicates 0.2 nucleotide substitutions per site.

To investigate the origins of *G. apicola* MPI, the phylogenetic relationships of *manA* were reconstructed (Fig. 4). We found that sequences from *G. apicola* formed a single clade that clustered most closely with sequences from other *Orbales* (*F. perrara* and *Orbus hercynius*) and are more distantly related to sequences from other gammaproteobacteria (Fig. 4). The only two sequences obtained from bumble bee species (*Bombus griseocollis* and *Bombus impatiens*) form a monophyletic group that is nested within the larger clade representing strains from honey bees. Phylogenies of the neighboring genes for PDH and RF1 also show nesting of sequences from bumble bee-derived isolates within those of honey bee-derived isolates (see Fig. S2 in the supplemental material); the same phylogenetic relationship is evident from the whole-genome tree (Fig. 1). Altogether, these results indicate that the PDH, RF1, and MPI genes were present in the common ancestor of *G. apicola* strains and that the absence of *manA* in certain strains is due to gene loss during evolution.

### Growth ability on mannose, xylose, arabinose, and rhamnose.

**FIG 5:**
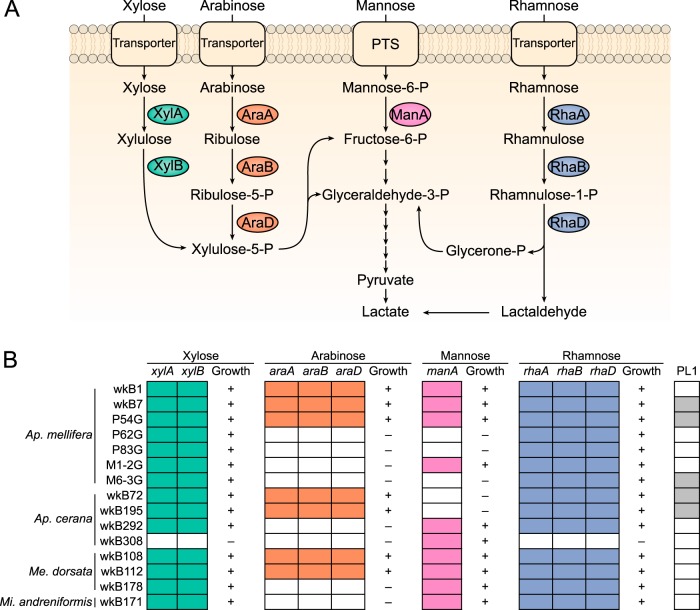
Variability in the presence of genes for sugar utilization among *G. apicola* strains. (A) Mannose, rhamnose, arabinose, and xylose metabolism pathways and related genes in *G. apicola*. (B) Presence of the genes related to the metabolism of the four sugars and the pectate lyase gene (PL1) in *G. apicola* strains isolated from honey bee guts. Colored boxes indicate gene presence, and white boxes indicate gene absence. The growth ability on the substrate is shown by plus (growth) and minus (no growth) signs.

In addition to mannose, other polysaccharide hydrolysates can be toxic to bee hosts (9). We screened for the genes encoding enzymes related to the catabolism of xylose (*xylA* [xylose isomerase] and *xylB* [xylose kinase]), arabinose (*araA* [arabinose isomerase], *araB* [ribulokinase], and *araD* [l-ribulose-5-P 4-epimerase]), and rhamnose (*rhaA* [rhamnose isomerase], *rhaB* [rhamnulose kinase], and *rhaD* [rhamnulose-1-phosphate aldolase]) (Fig. 5A), and we tested the ability of the *G. apicola* isolates to grow on different sugars.

*G. apicola* isolates were grown on a variety of monosaccharides (i.e., xylose, arabinose, mannose, and rhamnose) as the sole carbon and energy source. Since none of the *G. apicola* strains from bumble bee guts grew in the tested medium (see Materials and Methods) even with glucose and fructose, we only show the results for strains from honey bees (Fig. 5B). We found that growth abilities were consistent with the presence of the requisite genes for the catabolism of each sugar. Five strains grew on all four tested sugars. All of the strains from honey bees could grow on xylose and rhamnose, except strain wkB308 from *A. cerana*, which only grew on mannose. In contrast, the genes related to catabolizing all four sugars are underrepresented in the strains from bumble bees. Only *araABD* were detected from strains App2-1 and App2-10 from *Bombus appositus* (see Fig. S3 in the supplemental material), and only *manA* from strains Gris1-4 and Imp1-6 (Fig. 3; see also Fig. S3).

To determine their potential ability to degrade pectin and galacturonic acid, the main constituent of pectin, we also screened for the major pectate lyase (PL1) encoded in the honey bee gut metagenome (17) and genes related to galacturonic acid catabolism. The PL1 gene was detected in five genomes, and there is no clear correlation with monosaccharide degradation ability (Fig. 5B). All five genes in the pathway converting d-galacturonic acid to pyruvate and d-glyceraldehyde-3-phosphate, which have been described as active in *Escherichia coli* (25), are present in all *G. apicola* strains (data not shown), while the alternative oxidative pathway found in *Pseudomonas* (26) is totally absent from all *G. apicola* strains.

### Simultaneous sugar utilization.

To determine if the presence of mannose affects the growth of *G. apicola*, strains wkB1 and P62G from *A. mellifera* and strain wkB292 from *A. cerana* were grown on (i) a 1:1 mixture of glucose and fructose, which approximates the natural ratio in honey, (ii) a 1:1:1 mixture of glucose, fructose, and mannose, and (iii) mannose alone.

**FIG 6:**
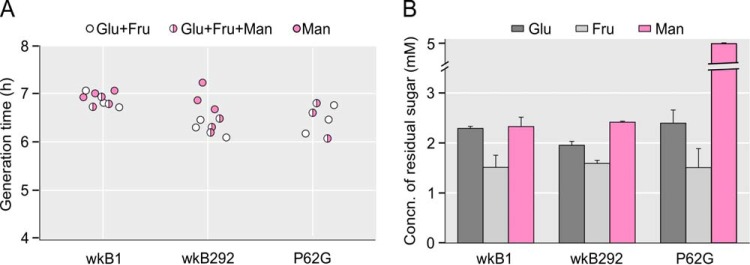
Sugar utilization by *G. apicola* strains. (A) *G. apicola* strains wkB1, wkB292, and P62G grew at approximately the same rates on the different sugar mixtures tested. Each circle represents the value for a batch culture. (B) Simultaneous utilization of sugars during the growth of *G. apicola* strains in batch cultures. Glucose (Glu), fructose (Fru), and mannose (Man) were added to cultures at the time of inoculation to a concentration of 5 mM. The concentrations of each sugar remaining after 24 h are shown (*n* = 3). Error bars show standard deviations.

On all substrates tested, all three strains typically grew to final cell densities of 0.5 × 10^8^ to 1.5 × 10^8^ cells/ml after 24 h at 35°C, with doubling times of 6.1 to 7.3 h (Fig. 6A). In the medium with all three sugars, strains wkB1 and wkB292 simultaneously utilized the individual sugars present (Fig. 6B). In all strains, fructose was consumed to the greatest extent. Although strain P62G cannot grow on mannose as it lacks MPI, the presence of equal amounts of mannose (5 mM) with glucose and fructose did not alter its growth; fructose and glucose were degraded to the same extent as in the other two strains, while all mannose added to the medium remained in the culture supernatant. This suggests that mannose was not transported into the cell through the PTS, despite the presence of a complete mannose PTS encoded in the genome. Altogether, these results indicate that *G. apicola* can utilize different sugars simultaneously and that the absence of MPI does not affect the growth rate, suggesting that mannose is not toxic to *G. apicola*, in contrast to what has been shown in its bee hosts.

## DISCUSSION

In this study, we document that one of the major bee gut symbionts, *G. apicola*, has the ability to metabolize mannose, xylose, arabinose, and rhamnose. These monosaccharides have been shown to reduce the life span of adult bees at low concentrations (11). Although the ability to metabolize these sugars varies among *G. apicola* strains, all strains tested from honey bee guts can utilize at least one of these sugars. Additionally, there is a strong correlation between the ability to grow on particular sugar substrates and the presence of the corresponding genes in catabolic pathways. Thus, we can predict with confidence the metabolic capabilities of strains from genomic sequence data.

The previous observations of sugar toxicity were based on bees that emerged naturally within hives and that presumably contained typical gut bacteria, so the contributions of gut symbionts to toxicity reduction were not assessed. *G. apicola* is acquired by all adult worker honey bees soon after their emergence within the colony, and this species comprises up to 39% of the bee gut microbiota (27). Our finding that many *G. apicola* strains can utilize certain sugars suggests that these gut symbionts may add to the energy budget of the host by helping to digest recalcitrant carbohydrates, as has been observed for bacteria in other insect guts (28) and in the human gut (1).

Although all *G. apicola* strains possess mannose family PTSs, including the *manO*-associated EII complex, the MPI enzyme is essential for mannose catabolism; in other bacteria, disruption of MPI can even repress growth on other carbon substrates (29). The mechanism of mannose toxicity in honey bees has been proposed to result from a deficiency of MPI. In bees, the ATP-driven phosphorylation of mannose to mannose-6-phosphate (mannose-6-P) is performed by hexokinase, and the imbalance of hexokinase and MPI expression leads to a competitive inhibition of glucose and fructose utilization when mannose is abundant (10). In a transcriptome data set from guts of *A. cerana* bees (30), the MPI transcript abundance was only ~6% of that of hexokinase transcripts. The result is an accumulation of intracellular mannose-6-P and ATP depletion due to the inefficiency of the bee host MPI in shunting mannose-6-P into glycolysis pathways (31).

We found that *G. apicola* lacking the gene encoding MPI, *manA*, is able to utilize glucose and fructose even when mannose is present in the medium, with no effect on growth. Thus, it seems that *G. apicola* does not experience the same mannose toxicity as its bee hosts. Our results suggest that this is due to mannose not being imported into *G. apicola* cells when *manA* is absent, despite the fact that all strains carry mannose family PTSs. How this is accomplished is unclear. A previous study of the *manO*-associated PTS EII complex in *Streptococcus thermophilus* also failed to observe cellular mannose uptake *in vivo* but detected mannose phosphorylation activity in cell-free experiments (32). Further gene expression studies will be required to unravel the regulatory mechanisms that allow *G. apicola* to suppress mannose import when mannose-6-P cannot be catabolized. It is likely that this metabolic flexibility permits the decoupling of mannose PTS and *manA* and the loss of *manA* in many *G. apicola* strains (Fig. 3).

Variability in carbohydrate utilization capabilities appears to be the norm among *G. apicola* strains. Carbohydrate metabolism genes constitute a large fraction of the accessory genome in six previously sequenced strains (16, 18). Overall, genes for sugar metabolism are highly represented in the honey bee gut community: a study of the bee gut metagenome documented that genes related to carbohydrate metabolism are specifically enriched compared to other gut-associated microbiomes (17), and genomic analyses of two abundant bee gut bacteria, *G. apicola* and *Lactobacillus* spp., uncovered a large proportion of genes in this functional group, particularly the mannose family PTSs (16, 33, 34), which are also shown to be preferentially transcribed in bee guts (35).

We showed that one mannose PTS with a *manO* regulator was acquired by the *Orbales* clade through horizontal gene transfer, likely from *Firmicutes* (Fig. 2). This PTS was shown to have undergone complex evolutionary events (23). This transfer is of particular interest here because it might allow the bee gut symbiont to digest toxic sugars. In *Orbales*, the *manO*-associated PTS was coupled to a gammaproteobacterial *manA* (Fig. 4), thus allowing mannose utilization; however, the *manA* gene was subsequently lost in many *G. apicola* strains, while the *manO*-associated PTS was retained. The comparison of gene arrangements in contigs containing *manA* reveals variation among different strains and supports the view that *manA* and other neighboring genes were present in the *G. apicola* ancestor and were gradually lost during strain diversification (Fig. 3). In four *G. apicola* strains from bumble bees, restriction-modification genes replace the missing genes. This is consistent with previous evidence that restriction-modification systems can behave as mobile genetic elements in the evolution of gut symbionts and can cause genome rearrangements (36).

Although almost identical in their 16S rRNA gene sequences, *G. apicola* strains exhibit extensive intraspecific variation in the presence of genes related to different sugar metabolism and growth abilities on different substrates (18). We found perfect correspondence between the predicted gene repertoires for sugar degradation in *G. apicola* genomes and the ability of strains to utilize the particular sugars in culture. Strains from the same host can differ in sugar metabolism ability, which is possibly due to the adaption to distinct metabolic niches. Notably, almost all *G. apicola* strains from bumble bees have lost the genes for the metabolism of the investigated sugars, which corroborates earlier suggestions that bumble bee *G. apicola* strains have fewer carbohydrate degradation capabilities than their honey bee counterparts (16). The diversification of *G. apicola* strains in different hosts is potentially shaped by the differing diets, longevities, and nest population sizes between honey bee and bumble bee species (37).

Bees have a carbohydrate-rich diet of nectar and pollen (38) and, probably, a high sugar concentration in their guts. Pectins and polysaccharides are present in the pollen wall, and some *G. apicola* strains possess pectate lyases and glycoside hydrolases, which can break down the pectin backbone and side chains (16, 17). We also found that some *G. apicola* strains have the PL1 gene; however, there is no apparent correlation between the ability to secrete enzymes for pectin degradation and the ability to utilize the constituent monosaccharides. Thus, pectin digestion and downstream sugar uptake and metabolism can be performed by different species or by different strains of the same species. Potentially, different hives have different profiles of *G. apicola* strains, with consequences for their ability to derive energy from pollen components and for their ability to resist the toxic effects of component sugars. Nevertheless, *in vivo* analyses of the ability of *G. apicola* strains to metabolize mannose in the bee gut will further characterize the effects of gut microbiota on bee health. High strain diversity within guts of individual bees or within hives could be beneficial by promoting nutrient availability, mitigating toxins, and improving immune response and microbiota stability, as has been suggested for human individuals with high gut microbiota richness (39, 40).

## MATERIALS AND METHODS

### Bee samples.

We collected honey bees (order *Hymenoptera*, family *Apidae*, genus *Apis*) comprising 4 species and bumble bees (genus *Bombus*) comprising 10 species belonging to different subgenera. The collection locations and dates of samples used in this study are shown in Table S1 in the supplemental material. The bees were identified based on their morphology. The dissected guts were either homogenized in 10 mM MgSO_4_ for immediate bacterial isolation or crushed in 19% glycerol and frozen directly after sampling.

### Isolation and cultivation of *G*. *apicola.*

Pure cultures of *G. apicola* strains were isolated from bee guts as previously described (41). Briefly, fresh gut homogenates or glycerol stocks were plated on heart infusion agar (HIA) supplemented with defibrinated sheep’s blood (5% [vol/vol] final concentration; Hardy Diagnostics). After 2 days of incubation at 37°C under a CO_2_-enriched atmosphere (5%), visible colonies were identified by sequencing of the 16S rRNA gene.

The ability of *G. apicola* strains to grow on different substrates was tested by cultivation in BYZ medium containing a buffered salts solution (BSS) (42) composed of (per liter) 0.2 g KH_2_PO_4_, 0.25 g NH_4_Cl, 0.5 g KCl, 0.15 g CaCl_2_ ⋅ 2H_2_O, 1.0 g NaCl, 0.62 g MgCl_2_ ⋅ 6H_2_O, 2.84 g Na_2_SO_4_, and 10 mM MOPS (morpholinepropanesulfonic acid), as well as 0.05% (wt/vol) yeast extract. The medium was adjusted to a final pH of 7.0. Glucose, arabinose, xylose, or rhamnose (10 mM final concentration) was added as a carbon substrate. Cultures were established by inoculating the medium with single colonies growing on HIA plates and were incubated at 35°C under 5% CO_2_.

The ability of *G. apicola* strains to utilize different substrates simultaneously was tested by culturing the strains in medium containing BSS supplemented with 5 mM each of glucose, fructose, and/or mannose. Growth was determined spectrophotometrically by following the increase in optical density at 600 nm. After 24 h of incubation, 1-ml aliquots of culture medium were sampled and centrifuged at 12,000 × *g* for 5 min, and the supernatant was diluted 1:100 with water (high-performance liquid chromatography [HPLC] grade; Fisher Chemical). The concentration of residual glucose, fructose, or mannose in the diluted culture medium was determined by using the d-mannose/d-fructose/d-glucose assay kit (Megazyme, Inc.).

### Genome sequencing and annotation.

Genomic DNA was extracted using phenol-chloroform and purified on DNeasy spin columns (Qiagen). Paired-end libraries of 2- to 4-kb insert sizes were constructed and sequenced on the Illumina MiSeq platform as described previously (16). The reads obtained were assembled with MaSuRCA version 3.1.0 (43). For strain wkB7, a complete genome was generated by sequencing on the PacBio RS II platform and assembly with the hierarchical genome assembly process (HGAP) method (Pacific Biosciences, Inc.); the assembly was closed and verified by mapping Illumina MiSeq reads onto the HGAP-generated contigs. All genomes were annotated with the RAST server (44). Annotated genomes were imported into Geneious version 9.1.4 (45) for visualization and further analysis. Pairwise average nucleotide identities were calculated using the pyani Python3 module (https://github.com/widdowquinn/pyani).

### Phylogenetic analysis.

To generate the whole-genome tree, we used a concatenated alignment of 225 single-copy genes shared among all *G. apicola* strains. Orthologous genes were identified using best bidirectional hit with USEARCH (46) and clustered using the Markov cluster algorithm (47). The shared single-copy genes were aligned with MAFFT version 7 (48). The alignments of the nucleotide sequences were then concatenated, and maximum-likelihood trees were inferred using RAxML version 8 with the GTRGAMMA nucleotide substitution model (49).

For the phylogenetic analysis of *manA* and the mannose family EII complex, corresponding nucleotide sequences from *G. apicola* and close hits in the NCBI GenBank nr database were codon aligned using MAFFT version 7 (48). After manual refinement of the alignments, maximum-likelihood trees were constructed using MEGA7 (50) with 1,000 bootstraps and the GTR+G+I model, which was tested to best describe the substitution pattern using MEGA7.

### Accession number(s).

All genome sequences of *G. apicola* strains isolated in this study have been deposited in the Whole Genome Shotgun projects at DDBJ/ENA/GenBank. The accession numbers are listed in Table S1.

## SUPPLEMENTAL MATERIAL

Figure S1  Presence (+) of 11 mannose family phosphotransferase systems with enzyme IID domains in *G. apicola* strains. Download Figure S1, PDF file, 0.1 MB

Figure S2  Maximum-likelihood trees of the concatenated sequences of three pyruvate dehydrogenase (PDH) components and peptide release factor 1 (PRF) of *G. apicola* strains and *F. perrara*. Download Figure S2, PDF file, 0.1 MB

Figure S3  Presence of the genes related to the metabolism of the four sugars and the pectate lyase gene (PL1) in *G. apicola* strains isolated from bumble bee guts. Download Figure S3, PDF file, 0.1 MB

Table S1  Genome features of the *G. apicola* strains isolated from the guts of honey bees and bumble bees. Table S1, PDF file, 0.1 MB
