# Supplementary material for: Metabolism of Toxic Sugars by Strains of the Bee Gut Symbiont Gilliamella apicola
Source: mBio. 2016 Nov 1;7(6):e01326-16. doi: 10.1128/mBio.01326-16 (PMC5090037; doi:10.1128/mBio.01326-16)
Supplement: Figure S1 — Presence (+) of 11 mannose family phosphotransferase systems with enzyme IID domains in G. apicola strains. Download [file mbo005163054sf1.pdf]

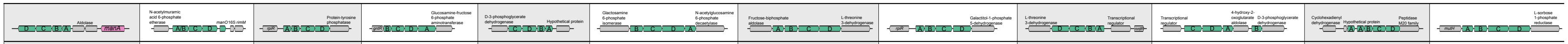

Apis	wkB1	+	+	+	+								+
	P54G	+	+	+	+								+
	wkB7	+	+	+	+		+						+
	M6-3G	+	+	+	+	+	+	+					
	M1-2G		+	+	+	+	+	+					
	P62G		+	+	+	+	+	+					
	P83G		+	+	+	+	+	+					
	wkB308		+		+		+						
	wkB292		+	+	+	+	+						
	wkB72		+	+	+	+							+
	wkB195		+	+	+	+							+
	wkB112		+						+				+
	wkB178		+									+	
	wkB108		+	+	+	+				+	+		
	wkB171		+	+	+	+	+						
Bombus	Gris1-4	+	+		+	+	+						
	Imp1-6	+	+		+	+	+						
	Imp1-1		+										
	Choc5-1		+			+							+
	GillExp13		+	+		+							
	App6-5		+										
	Occ4-3		+										
	Bif1-4		+										
	Bim1-2		+										

Fig. S1 The presence ("+") of 11 mannose-family phosphotransferase systems with Enzyme IID domains in *G. apicola* strains. These PTS may also function in the transport of sugars other than mannose, such as glucose, sorbose, fructose, and N-acetylglucosamine.
