# Supplementary material for: Metabolism of Toxic Sugars by Strains of the Bee Gut Symbiont Gilliamella apicola
Source: mBio. 2016 Nov 1;7(6):e01326-16. doi: 10.1128/mBio.01326-16 (PMC5090037; doi:10.1128/mBio.01326-16)
Supplement: Figure S2 — Maximum-likelihood trees of the concatenated sequences of three pyruvate dehydrogenase (PDH) components and peptide release factor 1 (PRF) of G. apicola strains and F. perrara. Download [file mbo005163054sf2.pdf]

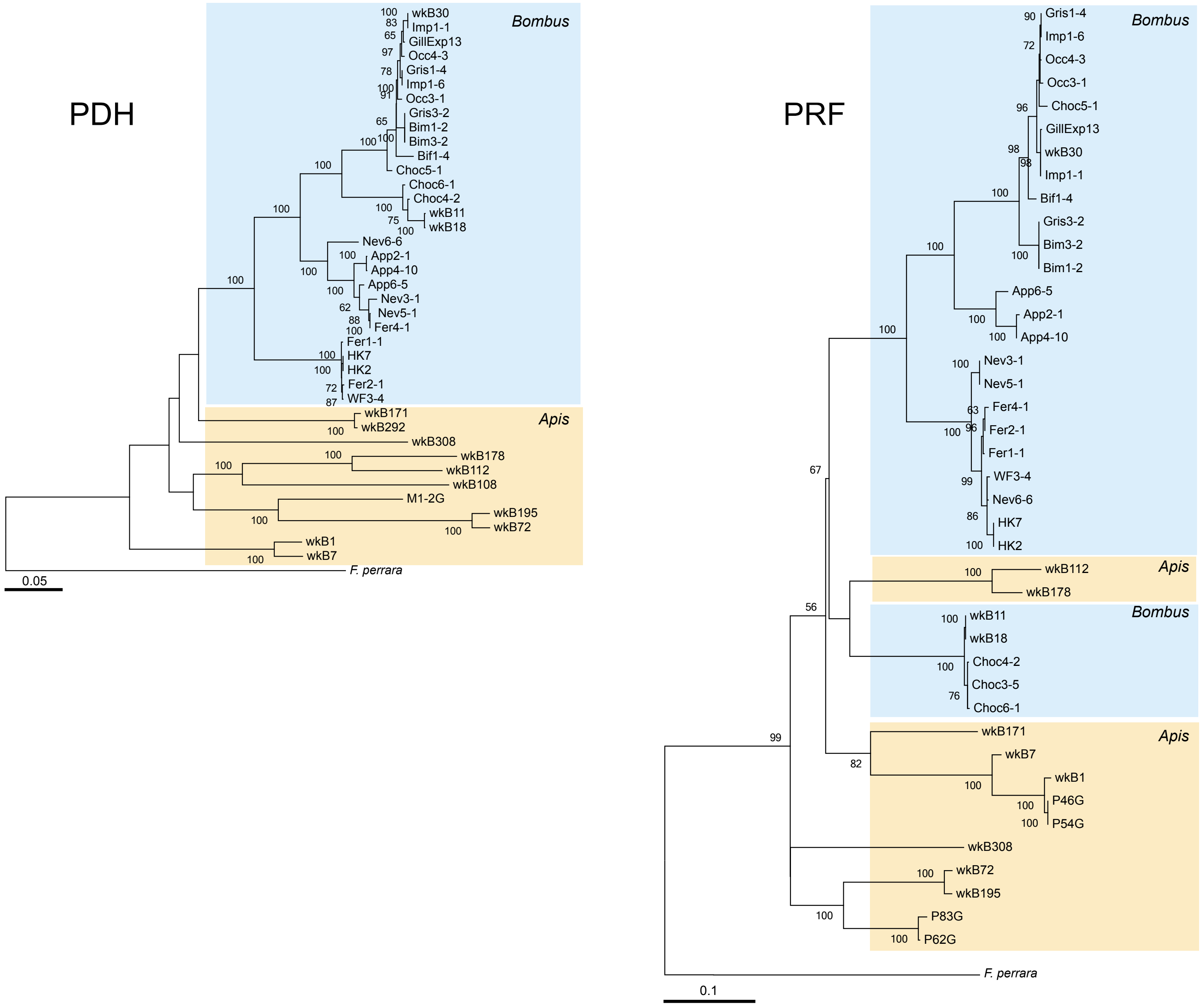

Fig. S2 Maximum-likelihood trees of the concatenated sequences of three pyruvate dehydrogenase components (PDH) and the peptide release factor (PRF) of *G. apicola* strains and *Frischella perrara*. The trees are based on nucleotide sequences and were rooted using homologs from *F. perrara*. Nodes with bootstrap values >50% are marked (1,000 replicates).
