# Supplementary material for: Metabolism of Toxic Sugars by Strains of the Bee Gut Symbiont Gilliamella apicola
Source: mBio. 2016 Nov 1;7(6):e01326-16. doi: 10.1128/mBio.01326-16 (PMC5090037; doi:10.1128/mBio.01326-16)
Supplement: Figure S3 — Presence of the genes related to the metabolism of the four sugars and the pectate lyase gene (PL1) in G. apicola strains isolated from bumble bee guts. Download [file mbo005163054sf3.pdf]

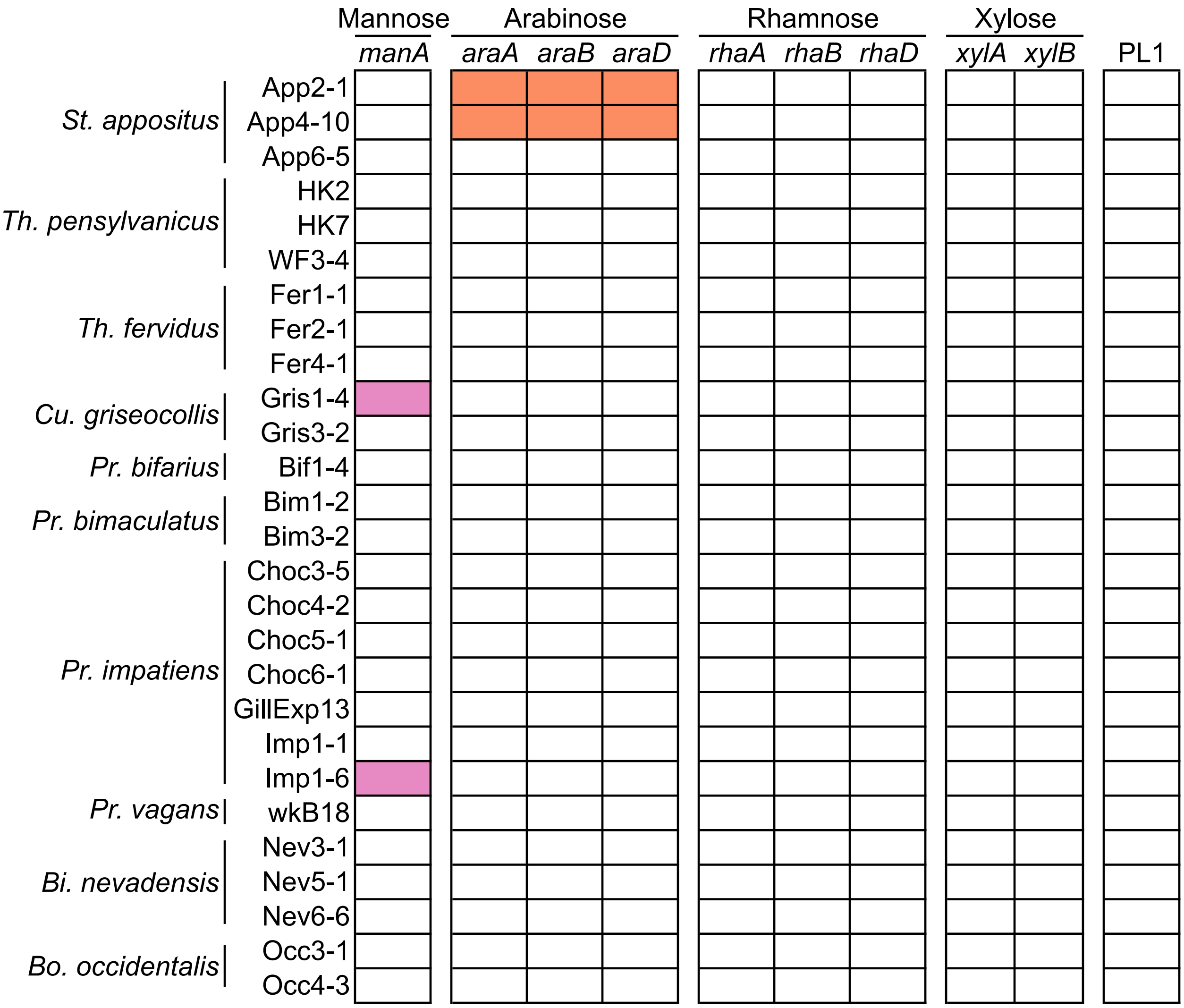

Fig. S3 The presence of the genes related to the metabolism of the four sugars and the pectate lyase gene (PL1) in *G. apicola* strains isolated from bumble bee guts. Colored boxes indicate gene presence, and white boxes indicate gene absence.
