# Supplementary material for: Metabolism of Toxic Sugars by Strains of the Bee Gut Symbiont Gilliamella apicola
Source: mBio. 2016 Nov 1;7(6):e01326-16. doi: 10.1128/mBio.01326-16 (PMC5090037; doi:10.1128/mBio.01326-16)
Supplement: Table S1 — Genome features of the G. apicola strains isolated from the guts of honey bees and bumble bees. [file mbo005163054st1.pdf]

Table S1. Genome features of the *Gilliamella apicola* strains isolated from honey bee and bumble bee guts.

Host genus	Host subgenus	Host species	Strain	Total size (bp)	No. of contigs	N50	Accession No.	16S rRNA similarity[a] (%)	Sequencing method	Collection location	Collection date
Apis	*Micrapis*	*Apis andreniformis*	wkB171	2,670,926	59	144,296	LYST00000000	99.0	Illumina MiSeq 2×300	Hort Park, Singapore	August, 2014
	Megapis	*Apis dorsata*	wkB108	3,076,170	23	77,698	LZGM00000000	98.1	Illumina MiSeq 2×300	University of Malaya, Kuala Lumpur, Malaysia	July, 2014
			wkB112	2,532,308	22	293,117	LZGL00000000	97.9	Illumina MiSeq 2×300	University of Malaya, Kuala Lumpur, Malaysia	July, 2014
			wkB178	2,369,030	16	588,434	LZGK00000000	98.9	Illumina MiSeq 2×300	Hort Park, Singapore	August, 2014
	Apis	*Apis cerana*	wkB72	2,570,834	79	213,610	LZEI00000000	99.0	Illumina MiSeq 2×300	Kampung Baru Kuning Selatan, Negeri Sembilan, Malaysia	July, 2014
			wkB195	2,500,996	38	101,351	LZGP00000000	99.0	Illumina MiSeq 2×300	Hort Park, Singapore	August, 2014
			wkB292	2,741,181	85	98,188	LZGO00000000	99.0	Illumina MiSeq 2×300	Hort Park, Singapore	August, 2014
			wkB308	2,737,107	46	195,029	LZGN00000000	97.2	Illumina MiSeq 2×300	Genting Highlands, Pahang, Malaysia	July, 2014
		Apis mellifera	P54G	3,105,460	56	129,582	LZGJ00000000	99.3	Illumina MiSeq 2×250	West Haven, CT, USA	September, 2011
			P46G	7,423,003	2,781	5,899	MCIV00000000	99.3	Illumina MiSeq 2×250	West Haven, CT, USA	September, 2011
			P62G	2,549,106	20	172,591	LZGI00000000	99.3	Illumina MiSeq 2×250	West Haven, CT, USA	September, 2011
			P83G	2,494,021	29	230,310	LZGH00000000	99.1	Illumina MiSeq 2×250	West Haven, CT, USA	July, 2014
			wkB7	2,901,642	1	2,901,642	LZGG00000000	99.6	PacBio RSII, Illumina MiSeq 2×250	West Haven, CT, USA	September, 2011
			M1-2G	2,388,823	10	342,893	LZGQ00000000	99.0	Illumina MiSeq 2×250	West Haven, CT, USA	May, 2011
			M6-3G	2,700,722	83	191,995	MCIU00000000	98.9	Illumina MiSeq 2×250	University of Texas, Austin, TX, USA	February, 2014
Bombus	*Subterraneobombus*	*Bombus appositus*	App2-1	2,418,433	73	71,484	LZGR00000000	98.1	Illumina MiSeq 2×300	East of Logan along Hwy 89, Utah, USA	July, 2014
			App4-10	2,434,386	53	145,820	LZGS00000000	98.1	Illumina MiSeq 2×300	East of Logan along Hwy 89, Utah, USA	July, 2014
			App6-5	2,633,935	106	66,665	LZGT00000000	98.6	Illumina MiSeq 2×300	East of Logan along Hwy 89, Utah, USA	July, 2014
	Thoracobombus	*Bombus pensylvanicus*	HK2	2,843,330	117	79,103	LZGV00000000	98.8	Illumina MiSeq 2×250	Brackenridge Field Lab, Austin, Texas, USA	June, 2013
			HK7	2,832,078	137	59,720	LZGU00000000	98.5	Illumina MiSeq 2×250	Brackenridge Field Lab, Austin, Texas, USA	June, 2013
			WF3-4	2,847,396	61	165,092	LZGW00000000	99.0	Illumina MiSeq 2×300	Lady Bird Johnson Wildflower Centre, Austin, Texas, USA	June, 2014
		Bombus fervidus	Fer1-1	2,741,337	118	44,896	LZGX00000000	98.9	Illumina MiSeq 2×300	Logan, Utah, USA	July, 2014
			Fer2-1	2,611,703	207	24,516	LZGY00000000	99.0	Illumina MiSeq 2×300	Logan, Utah, USA	July, 2014
			Fer4-1	2,520,134	360	12,144	LZGZ00000000	98.7	Illumina MiSeq 2×300	Logan, Utah, USA	July, 2014
	Cullumanobombus	*Bombus griseocollis*	Gris1-4	2,358,864	90	58,267	LZHA00000000	98.6	Illumina MiSeq 2×250	New Haven, CT, USA	June, 2013
			Gris3-2	2,552,527	89	61,124	LZHB00000000	98.6	Illumina MiSeq 2×300	New Haven, CT, USA	June, 2013
	Pyrobombus	*Bombus bifarius*	Bif1-4	2,551,594	98	176,528	LZHC00000000	98.8	Illumina MiSeq 2×300	East of Logan along Hwy 89, Utah, USA	July, 2014
		Bombus bimaculatus	Bim1-2	2,537,475	107	71,558	LZHD00000000	98.6	Illumina MiSeq 2×250	New Haven, CT, USA	June, 2013
			Bim3-2	2,592,778	177	37,453	LZHE00000000	98.6	Illumina MiSeq 2×300	New Haven, CT, USA	June, 2013
		Bombus impatiens	Choc3-5	2,193,011	89	74,181	LZHF00000000	97.2	Illumina MiSeq 2×250	New Haven, CT, USA	August, 2012
			Choc4-2	2,245,186	78	79,959	LZHG00000000	97.2	Illumina MiSeq 2×250	New Haven, CT, USA	August, 2012
			Choc5-1	2,628,355	125	66,703	LZHH00000000	97.2	Illumina MiSeq 2×250	New Haven, CT, USA	August, 2012
			Choc6-1	2,180,622	68	105,607	LZHI00000000	96.2	Illumina MiSeq 2×250	New Haven, CT, USA	August, 2012
			GillExp13	2,392,232	163	33,677	LZHJ00000000	98.7	Illumina MiSeq 2×250	New Haven, CT, USA	August, 2012
			Imp1-1	2,359,762	141	34,994	LZHK00000000	98.7	Illumina MiSeq 2×250	New Haven, CT, USA	June, 2013
			Imp1-6	2,269,603	91	45,049	LZHL00000000	98.8	Illumina MiSeq 2×250	New Haven, CT, USA	June, 2013
		Bombus vagans	wkB18	2,371,993	25	67,286	LZHM00000000	97.2	Illumina MiSeq 2×250	New Haven, CT, USA	June, 2011
	Bombias	*Bombus nevadensis*	Nev3-1	2,857,512	93	106,933	LZHN00000000	98.8	Illumina MiSeq 2×300	East of Logan along Hwy 89, Utah, USA	July, 2014
			Nev5-1	2,713,492	94	74,914	LZHO00000000	98.9	Illumina MiSeq 2×300	East of Logan along Hwy 89, Utah, USA	July, 2014
			Nev6-6	2,689,628	103	70,613	LZHP00000000	99.4	Illumina MiSeq 2×300	East of Logan along Hwy 89, Utah, USA	July, 2014
	Bombus	*Bombus occidentalis*	Occ3-1	2,232,791	36	101,835	LZHQ00000000	98.8	Illumina MiSeq 2×300	East of Logan along Hwy 89, Utah, USA	July, 2014
			Occ4-3	2,686,218	124	56,684	LZHR00000000	98.6	Illumina MiSeq 2×300	East of Logan along Hwy 89, Utah, USA	July, 2014

[a]The 16S rRNA sequences of *G. apicola* strains were compared to that of strain wkB1.
